# Comparative effectiveness of school-based obesity prevention programs for children and adolescents: a systematic review and network meta-analysis

**DOI:** 10.3389/fpubh.2024.1504279

**Published:** 2024-12-17

**Authors:** Mohamed A. Hassan, Daniel J. McDonough, Suryeon Ryu, Wanjiang Zhou, John Oginni, Zan Gao

**Affiliations:** ^1^Department of Methods and Curriculum, Sports Science College, Helwan University, Cairo, Egypt; ^2^School of Public Health, Division of Epidemiology and Community Health University of Minnesota-Twin Cities, Minneapolis, MN, United States; ^3^Department of Kinesiology, Recreation, and Sport Studies, The University of Tennessee, Knoxville, TN, United States; ^4^School of Kinesiology, University of Minnesota-Twin Cities, Minneapolis, MN, United States

**Keywords:** physical activity, body mass index, body composition, anthropometry, diet, intervention

## Abstract

**Introduction:**

While many randomized controlled trials (RCTs) have demonstrated the positive effects of school-based programs in reducing body fat among children and teenagers, there is no conclusive evidence to indicate that one approach is superior to another, largely due to the lack of direct and indirect comparisons. This study evaluated the relative effectiveness of various school-based obesity prevention initiatives in improving body mass index (BMI) among children and adolescents using network meta-analysis.

**Methods:**

Searches included four databases focusing on articles published in English between the years 2002 and 2024. The primary outcomes were the BMI and BMI z-scores (BMIz) (kg/m^2^). The mean differences (MDs) for each outcome were calculated before and after treatment. The current systematic review synthesized 53 RCTs with a sample of 68,489 children and adolescents.

**Results:**

The results illustrated that the physical activity (PA) only arm was the most effective intervention in improving BMI (MD: −0.42, 95% credible interval (Crl) −0.79, −0.07; *p* = 0.02), while the multiple-component intervention was the most effective in improving BMIz (MD: −0.08, 95% Crl: −0.16, −0.01; *p* = 0.03). Inversely, PA and another component arm were the least effective interventions in improving BMI (MD: 0.64, 95% Crl: −0.23, 1.53; *p* = 0.15). In addition, diet and nutrition only arm was the least effective intervention in improving BMIz (MD: 0.09, 95% Crl: −0.11, 0.28; *p* = 0.36).

**Discussion:**

In conclusion, both PA-only and multiple-component arms are effective intervention tools/strategies for reducing BMI-related outcomes. However, further large-scale, well-designed studies are needed to investigate the elements of multiple-component arms.

**Systematic review registration:**

https://www.crd.york.ac.uk/PROSPERO/ identifier CRD42021234742.

## 1 Introduction

Childhood obesity, defined as having a body mass index (BMI) at or above the 95th percentile for a child’s age and gender (1), remains a significant public health challenge, with global prevalence increasing by approximately 50% in recent years (2). Immediate health concerns associated with childhood obesity include the development of cardiometabolic risk factors, respiratory and skeletal issues, and mental health conditions (3–5). The long-term risks are well-documented in epidemiological studies (6), which show that childhood obesity often persists into adulthood. Adult obesity is strongly linked to the onset of non-communicable diseases such as heart disease, type 2 diabetes, and certain cancers (7). Moreover, obesity and its related complications place a substantial economic burden on healthcare systems worldwide, driven by both direct medical expenses and indirect costs (8). Although the causes of obesity are multifactorial (5), its underlying mechanism is a sustained positive energy balance, resulting in progressive weight gain over time (5, 9).

Currently, evidence supporting the efficacy and safety of pharmacotherapy or bariatric surgery for weight loss in children with obesity remains limited (5, 10). Concerns have been raised about the small number of Food and Drug Administration (FDA)-approved anti-obesity medications available for pediatric populations, in contrast to the broader range of options for adults (11, 12). While bariatric surgery has shown promising results in treating obesity, there are significant concerns regarding post-surgical outcomes. Studies have highlighted the risks associated with the need for repeat surgeries due to weight regain, as well as the potential necessity of combining bariatric procedures with short-or long-term weight loss medications for some patients (13, 14). Unlike genetic and environmental risk factors for obesity, behavioral factors are largely modifiable (15, 16). Consequently, the primary strategy for treating and preventing pediatric obesity focuses on interventions that promote healthier behaviors to improve body weight regulation (17).

Within the framework of energy balance (9), the most modifiable behaviors are dietary intake (caloric consumption) and physical activity (PA) (caloric expenditure through movement, excluding resting energy expenditure and the thermic effect of food) (9). Recognizing this, the World Health Organization (WHO) and other organizations (15–17) have advocated for interventions aimed at modifying health behaviors by simultaneously reducing energy intake and increasing PA to regulate body weight in children. Given that children spend approximately half their day at school and consume approximately 50% of their daily caloric intake there, schools are a critical setting for implementing obesity prevention programs. Unlike home-or community-based interventions, school-based programs leverage existing infrastructure, allowing for efficient student engagement without significantly altering their daily routines or lifestyles (18).

Caloric consumption in educational settings is influenced by the food landscape both within schools (e.g., vending machines and food kiosks) and in their surrounding areas (e.g., fast-food outlets and grocery stores), as well as the policies supporting these environments. These factors have been linked to unhealthy dietary choices and higher BMI levels in children (19–24). Evidence shows that over the past two decades, the school food environment has significantly contributed to a decline in children’s consumption of unprocessed and minimally processed foods, alongside a marked increase in the intake of ultra-processed foods and high-calorie beverages, such as sugar-sweetened drinks (21, 22, 25–27). Technological advancements in food preparation and processing have made ultra-processed foods nutrient-poor, calorie-dense, and hyperpalatable (28, 29). Similarly, sugar-sweetened beverages, which have minimal impact on satiety, are hyperpalatable and often contribute substantially to children’s daily caloric intake (27). The widespread availability of these highly palatable foods and beverages, both in and around schools, capitalizes on innate human taste preferences for salt, sugar, and fat (30). Their frequent overconsumption fosters an obesogenic energy imbalance, exacerbating the risk of childhood obesity.

PA levels in schools are often insufficient to offset the excessive caloric intake associated with children’s dietary habits (31–34). Physical education classes frequently fail to sustain moderate-to-vigorous physical activity (MVPA) for the recommended minimum of 50% of class time. Moreover, children with higher weight status tend to engage less in MVPA during various segments of the school day (35, 36). Outside of school, increased screen time, reduced active transportation, and lower participation in leisure-time physical activities have further contributed to the global rise in childhood physical inactivity and sedentary behavior over recent decades (5, 37–39). The combination of excessive caloric intake, inadequate PA, and a genetic predisposition to store body fat has created an urgent need for public health interventions. Addressing the childhood obesity crisis requires the implementation of school-based programs that promote behaviors supportive of maintaining a healthy body weight (40).

Several school-based obesity prevention interventions have been shown to effectively reduce children’s weight-related outcomes in randomized controlled trials (RCTs) (41–44). However, challenges remain in translating and disseminating these findings into widespread, effective obesity prevention programs. A key issue is the lack of empirical evidence demonstrating the superiority of one intervention over another, as direct and indirect comparisons are often absent (41). Additionally, the data are mixed regarding the relative effectiveness of single-component versus multicomponent interventions (21). To address these gaps, we conducted a comprehensive systematic review of existing literature to identify RCTs evaluating the impact of school-based obesity prevention programs on children’s weight-related outcomes. This was followed by a network meta-analysis (NMA) to simultaneously assess the relative effectiveness of various intervention approaches compared to each other and to control groups. The findings offer valuable insights for policymakers and stakeholders at local, state, and federal levels, providing evidence to help identify the most effective school-based strategies for improving weight-related outcomes in children.

## 2 Methods

This study followed the guidelines outlined in the Preferred Reporting Items for Systematic Reviews and Meta-Analyses (PRISMA) extension statement for NMAs (45) and was registered with PROSPERO (CRD42021234742). As the analysis utilized previously published data and did not include individual participant data, institutional review board approval was not required.

### 2.1 Eligibility criteria and outcomes

The eligibility criteria were defined *a priori* using the population, intervention, comparators, outcomes, and setting (PICOS) framework (45). This review synthesized RCTs that evaluated school-based obesity prevention programs among school-aged children [6–12 years (5)]. Studies were required to have a minimum duration of one school year and to assess a bodyweight-related outcome, specifically BMI and/or BMI z-scores (BMIz) (kg/m^2^). To streamline the analysis and based on evidence that language restrictions do not consistently bias the results of quantitative syntheses (46), only studies published in English were included. Excluded studies were those that were not RCTs, were conducted outside of school settings, had a duration of less than one school year, and/or were not published in English.

### 2.2 Search strategy

**Figure 1:**
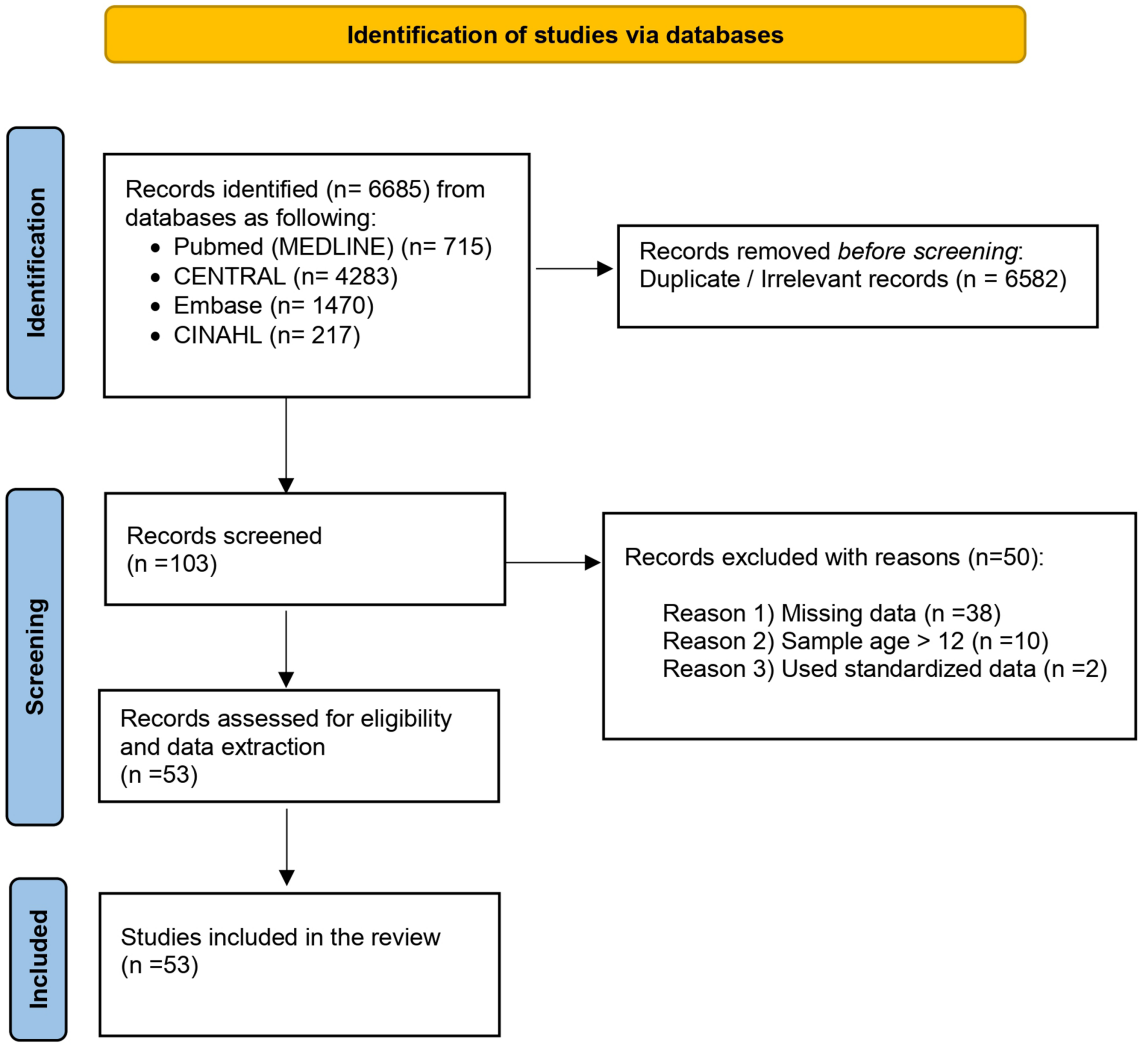
PRISMA flow chart for systematic reviews.

A systematic search was conducted across the databases MEDLINE (via PubMed), Embase, the Cochrane Central Register of Controlled Trials (CENTRAL), and CINAHL from inception through 10 September 2024. The search strategy, detailed in Figure 1, utilized a combination of medical subject headings (MeSH) and relevant keywords, including “physical activity,” “exercise,” “obesity prevention,” “nutrition,” “diet,” “multiple component,” and “adiposity”; (ii) “body mass index,” “anthropometrics,” “weight loss,” “BMI,” “BMIz,” “randomized controlled trials,” “school-based intervention,” “school children,” and “school program.” Examples of database search queries are provided in Supplementary File S1. Additionally, the researchers manually reviewed the reference lists of related systematic reviews and meta-analyses to identify any studies that might have been missed in the initial search. Three researchers (D.M., S.R., and W.Z.) independently screened titles, abstracts, and full-text articles to determine eligibility. Full-text articles of relevant RCTs were selected and evaluated for inclusion. Any discrepancies in opinion among the three researchers were resolved by a fourth author (Z.G.).

### 2.3 Screening and data extraction

Two independent reviewers (M.H. and J.O.) conducted an initial screening of studies by evaluating titles and abstracts. Studies that met the predefined criteria underwent a full-text review to confirm eligibility. Any disagreements between the reviewers were discussed and resolved with input from a third reviewer (Z.G.) to achieve consensus. Data from eligible studies were then independently extracted by the two reviewers using Microsoft Excel (Version 16.44 for Mac; Microsoft, Redmond, WA, United States). Any discrepancies in data extraction were resolved through consultation with the third reviewer.

The extracted data included the first author’s last name, publication year, study location, group sample sizes, gender distribution, mean age with standard deviation (SD), type and duration of the intervention, and outcome measures (see Supplementary File S2). The primary outcomes of interest were the mean change (SD_change_) from baseline to post-intervention. If these were not reported, baseline and post-intervention means and (SDs) were extracted, and the mean change was calculated as the difference, with the SD_change_ derived from pooled baseline and post-intervention variances. When only standard errors, 95% confidence intervals, *p*-values, or t-statistics were available, SDs were calculated accordingly.

For studies with multiple follow-up points, only the initial post-intervention data were extracted to focus on the immediate effects of the intervention rather than long-term outcomes. When available, intent-to-treat (ITT) analyses were prioritized, as they provide a more accurate representation of real-world scenarios where participants may not fully adhere to the intervention protocol.

### 2.4 Intervention categories

To identify the most effective health behavior change interventions using NMA (45), this study avoided grouping intervention types and instead evaluated six distinct interventions (comparators) against each other and a control group for their effectiveness in reducing bodyweight-related outcomes:

1. Control (usual care): Participants received no intervention beyond standard practices.
2. PA only: Participants received a school-based PA promotion intervention.
3. Diet and N only: Participants received a school-based diet and nutrition intervention.
4. PA and another component: Participants received a school-based PA promotion intervention combined with an additional component such as self-esteem instruction, education, or parent engagement.
5. PA and Diet and Nutrition (PA and Diet and N): Participants received a school-based intervention incorporating PA promotion, dietary education, and a nutritional program.
6. Multiple Components: Participants received interventions involving four or more components, such as PA, education, nutrition, lifestyle changes, parent engagement, and social media integration.

### 2.5 Risk of bias and quality of evidence

The risk of bias for the included studies was assessed by two researchers (S.R. and W.Z.) using the Cochrane risk of bias (RoB 2) assessment tool (47). The evaluation covered six domains: selection bias, performance bias, detection bias, attrition bias, reporting bias, and other potential sources of bias. Any disagreements between the two researchers were resolved through consultation with a third author (Z.G.). The detailed results are provided in Supplementary File S3.

### 2.6 Statistical analysis

In health behavior change research involving multiple intervention strategies, NMA enables the pooling of results from both direct and indirect evidence while preserving the advantages of randomized, within-trial comparisons (48). The transitivity assumption was assessed to ensure that the distribution of effect modifiers (e.g., sex and age) across studies supported reliable indirect comparisons (49). Once an even distribution of effect modifiers was confirmed and the transitivity assumption held, the NMAs were conducted using R Studio (version 2021.09.0, The R Foundation) and the BUGSnet package. This package adheres to the PRISMA, ISPOR-AMCP-NPC, and NICE-DSU guidelines, using a Bayesian approach with a burn-in of 50,000 iterations, followed by 100,000 iterations and 10,000 adaptations.

A random-effects model was used, and the analyses were performed with Markov Chain Monte Carlo (MCMC) simulations utilizing vague priors. Network geometry was assessed through network plots, with interpretation methods detailed in previous studies (50, 51). Model fit was evaluated using leverage plots, total residual deviance, and deviance information criterion (DIC). Forest plots and league plots were used to present network estimates for various comparisons. Intervention rankings were generated using surface under the cumulative ranking curve (SUCRA) plots (52).

It is important to interpret SUCRA values cautiously, as they may vary across outcomes for the same intervention, and such variations could be due to chance. SUCRA values should be considered alongside the quality of evidence, as they do not reflect the magnitude of outcome differences between the two interventions (52).

The results were not dichotomized as statistically significant or not; instead, they were presented with credible interval (CrI) to enable health practitioners to interpret the range of potential effects (53, 54). Specifically, comparative mean differences (MDs) were reported along with their associated 95% CrI, with the 2.5 and 97.5% quantiles serving as the lower and upper bounds, respectively.

## 3 Results

### 3.1 Search results and study characteristics

This NMA comprehensively synthesized data from 53 studies, including a total of 68,489 participants with a mean age of 9.40 years. Among them, 35,192 participants (51%) were assigned to intervention groups, while 33,297 (49%) were in control groups. Geographically, the studies represented 18 countries, with the following distribution:

- 14 studies (26%) from the USA (55–68).
- 5 studies each from Australia (69–73), China (74–78), and Spain (79–83).
- 3 studies each from the UK (84–86), Italy (87–89), and the Netherlands (90–92).
- 2 studies each from France (93, 94), Germany (95, 96), Greece (97, 98), and Switzerland (99, 100).
- 1 study each from Chile (101), Iceland (102), Ireland (103), Mexico (104), New Zealand (105), Norway (106), and Portugal (107).

The publication years ranged from 2002 to 2023, with 28 studies (50%) published in the last 10 years. Regarding intervention types:

- 12 studies focused on PA only (55, 58, 62, 64, 74, 79, 87, 93, 94, 99, 103, 105).
- 2 studies targeted Diet and N only (84, 89).
- 3 studies examined PA and another component (66, 86, 95).
- 9 studies focused on PA and Diet and N (60, 61, 68, 73, 78, 88, 96, 101, 104).
- 27 studies implemented multiple-component interventions (56, 57, 59, 63, 65, 67, 69–72, 75–77, 80–83, 85, 90–92, 97, 98, 100, 102, 106, 107).

### 3.2 Risk of bias assessment and quality of studies

The quality of the included studies was evaluated using the Cochrane RoB 2 assessment, with the results detailed in Supplementary File S2. Among the studies, 87% clearly described the process of random sequence generation. Regarding allocation concealment, only four studies (7%) were identified as having a high risk of bias. For blinding of participants and personnel, 13 out of 53 studies exhibited a high risk of performance bias, while 23 studies provided unclear reports on blinding for either participants or personnel. Similarly, for the blinding of outcome assessment, 36% of the studies demonstrated a low risk of bias, while the remaining studies presented either high or unclear risks. Only a small proportion of studies (7%) showed unclear or high risk of bias in addressing incomplete outcome data, minimizing the need for calculations to account for missing data. Finally, all studies reported the expected outcomes (BMI and/or BMIz) required for this NMA, indicating no or low risk of bias in the selective reporting domain.

### 3.3 Network geometry

**Figure 2:**
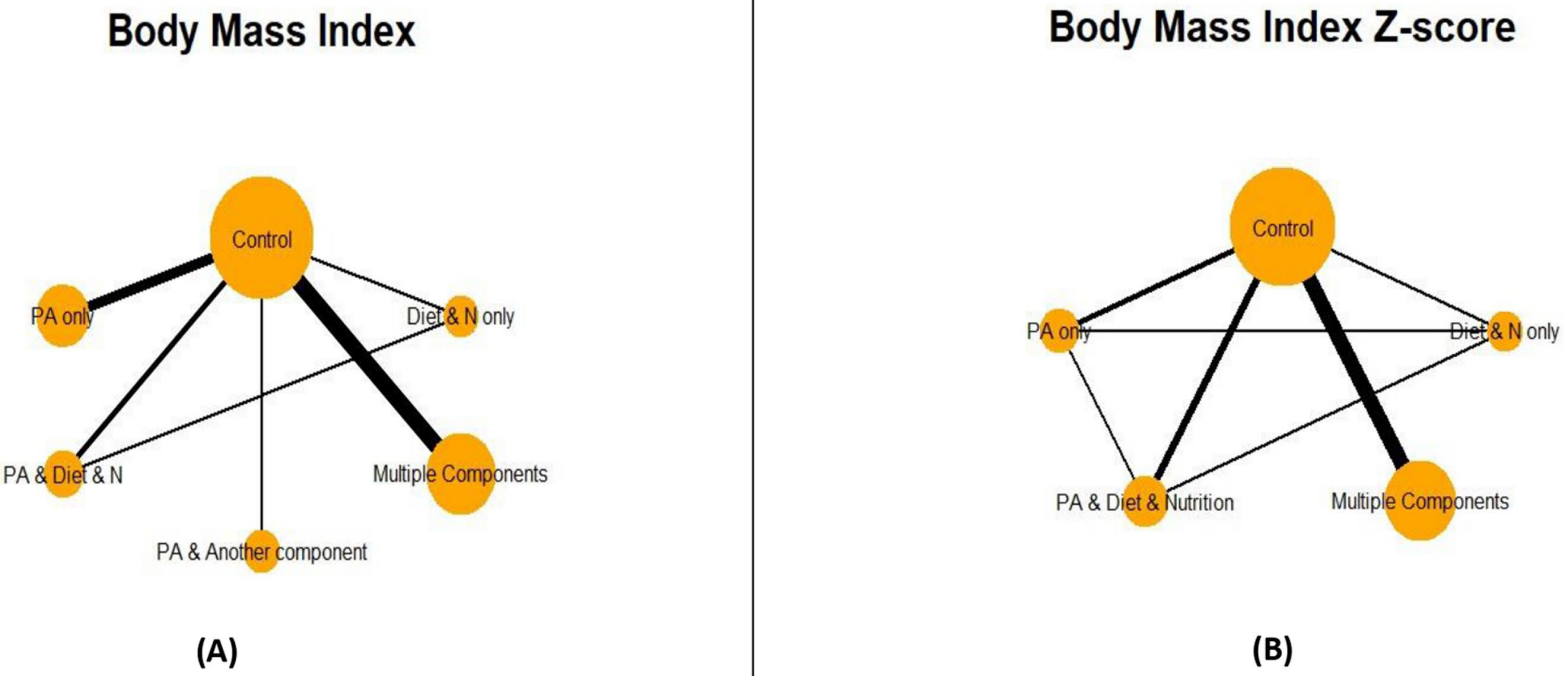
Network plot for body mass index **(A)** and body mass index *Z*-score **(B)**. PA = Physical Activity; *N*=Nutrition.

To detect all possible direct comparisons between treatments, network plots were generated to represent both BMI and BMIz outcomes, as shown in Figures 2A,B, respectively. In Figure 2A, closed loops were identified among the control group, Diet and N only, and PA and Diet and N interventions. Similarly, in Figure 2B, closed loops were observed among the control group, PA only, Diet and N only, and PA and Diet and N interventions. Closed loops indicate direct comparisons involving more than two interventions. Among all comparators, the control group and multiple-component interventions were the largest and of comparable size relative to the other comparators. Additionally, the thickest edge in the network plots represents the direct comparison between the control group and multiple-component interventions, highlighting their significant interaction.

### 3.4 Network meta-analysis

#### 3.4.1 Model fit

**Figure 3:**
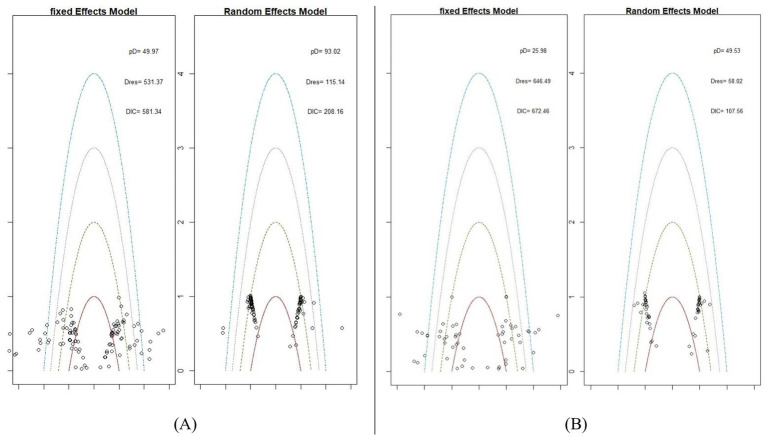
Leverage plots and DIC for fixed and random effects models for BMI **(A)** and BMIz **(B)**.

To select the appropriate model for the NMA, two models were tested for each outcome: a fixed-effect model and a random-effect model. Figure 3 highlights the identification of potential outliers. As shown in Figures 3A,B, three key metrics were considered: the effective number of parameters (pD), total residual deviance (Dres), and DIC. These values collectively informed the choice of the most suitable model for the network. Based on the data presented in Figures 3A,B, the random-effects model was selected for both BMI and BMIz. This decision was supported by the random-effects model showing fewer outliers and lower DIC values, indicating a better fit for the data.

#### 3.4.2 Consistency

**Figure 4:**
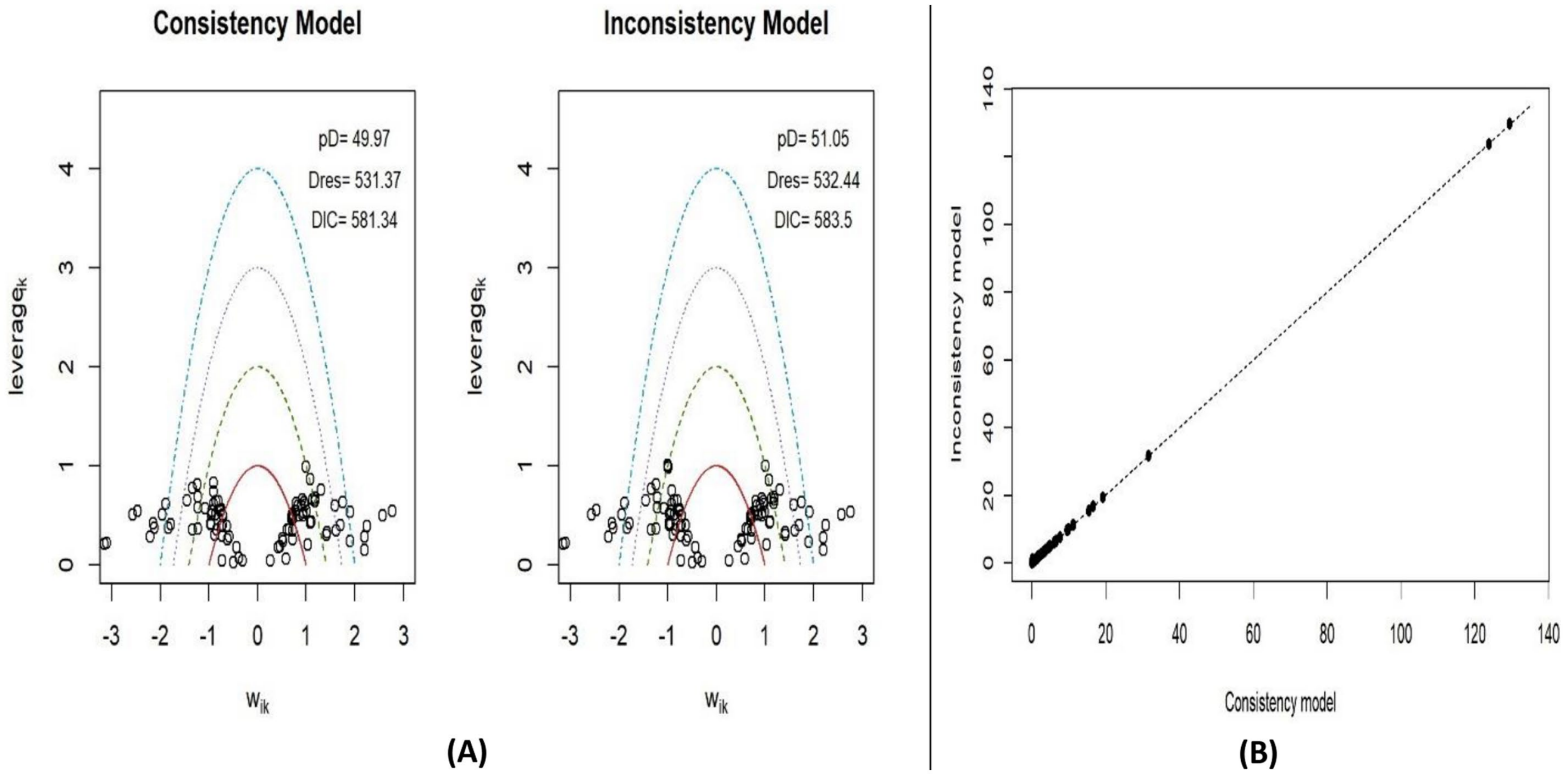
Leverage plots and DIC for consistency and inconsistency models for BMI **(A)** and plot of the posterior mean deviance of inconsistency model against consistency model for BMI **(B)**.

**Figure 5:**
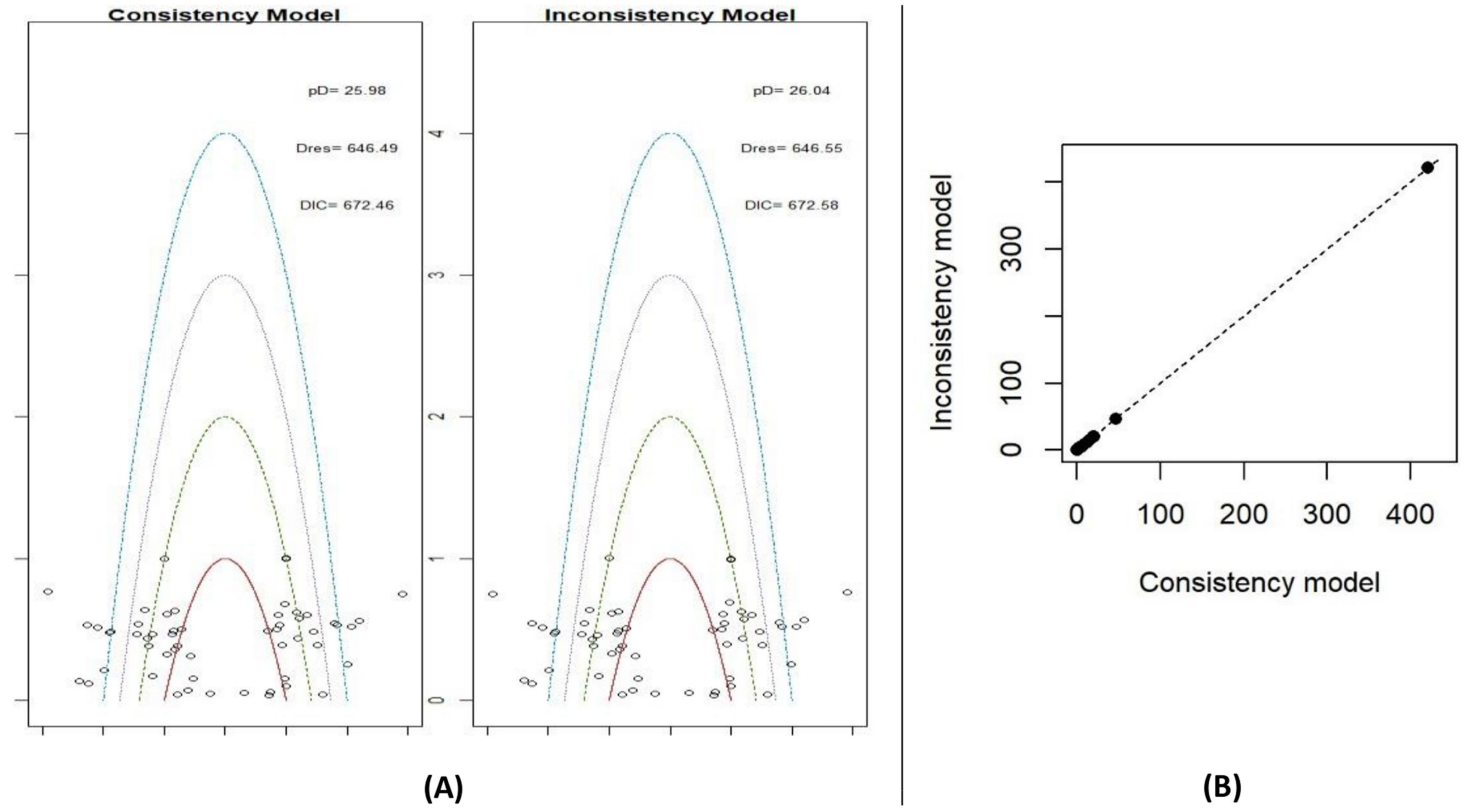
Leverage plots and DIC for consistency and inconsistency models for BMIz **(A)** and plot of the posterior mean deviance of inconsistency model against consistency model for BMIz **(B)**.

The assumption of consistency is a fundamental component of NMA. In simple terms, it ensures there is no significant discrepancy between direct and indirect comparisons across studies, thereby confirming the network’s consistency. To evaluate consistency in this NMA, two models were used: a consistency model and an inconsistency model. Model fit comparisons were assessed, and the posterior mean deviance of each combined model was plotted to visualize leverage points (Figure 4). For BMI, as shown in Figure 4A, the DIC values were lower in the consistency model. Additionally, the leverage values were more tightly clustered around zero in Figure 4B, indicating agreement between the two models and reducing the likelihood of inconsistency within the network. Similarly, the BMIz consistency model also exhibited lower DIC values (Figure 5), further supporting the consistency assumption for both BMI and BMIz outcomes in this NMA.

#### 3.4.3 Treatment ranking

**Figure 6:**
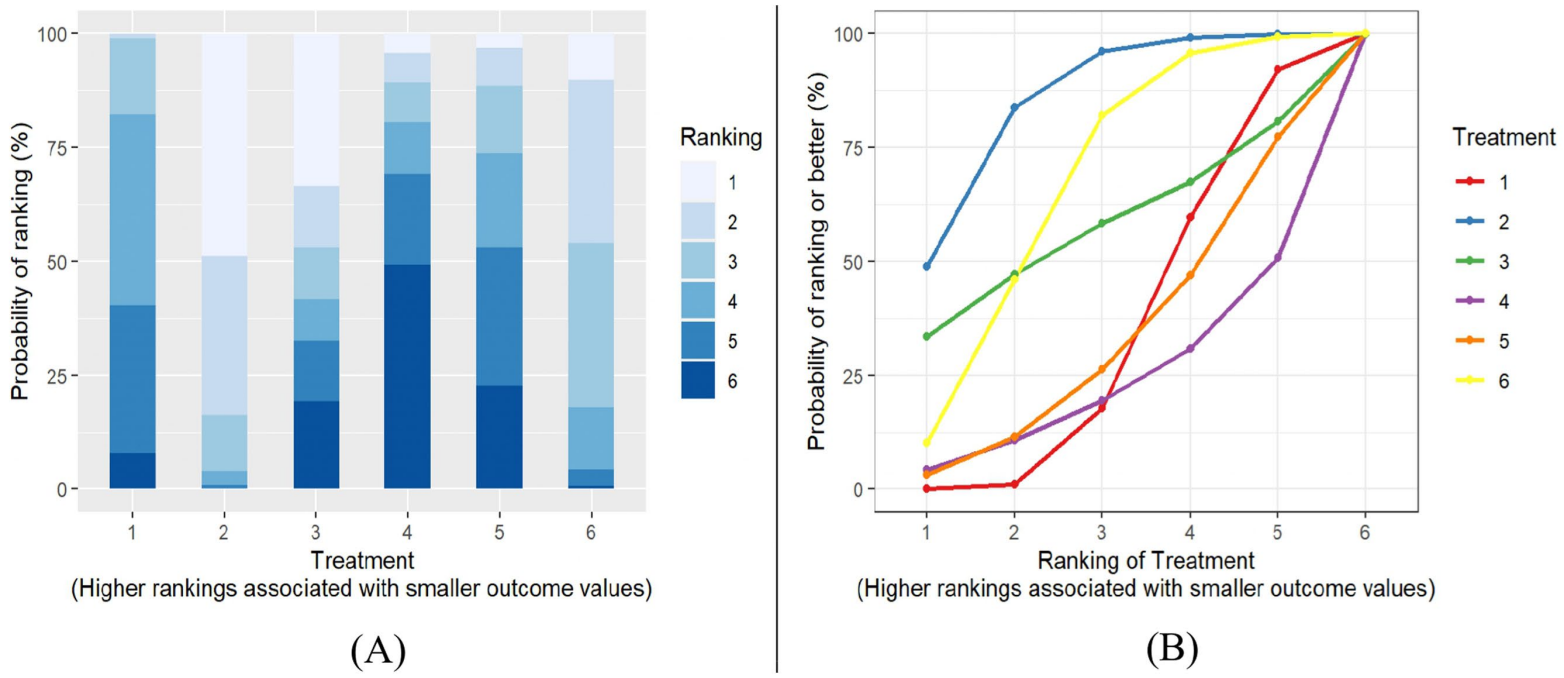
BMI Plot of treatment rank probabilities **(A)**, BMI SUCRA plot **(B)**. Treatments (1) control group; (2) PA only; (3) diet & only; (4) PA & another component; (5) PA & diet & N; (6) multiple components.

**Figure 7:**
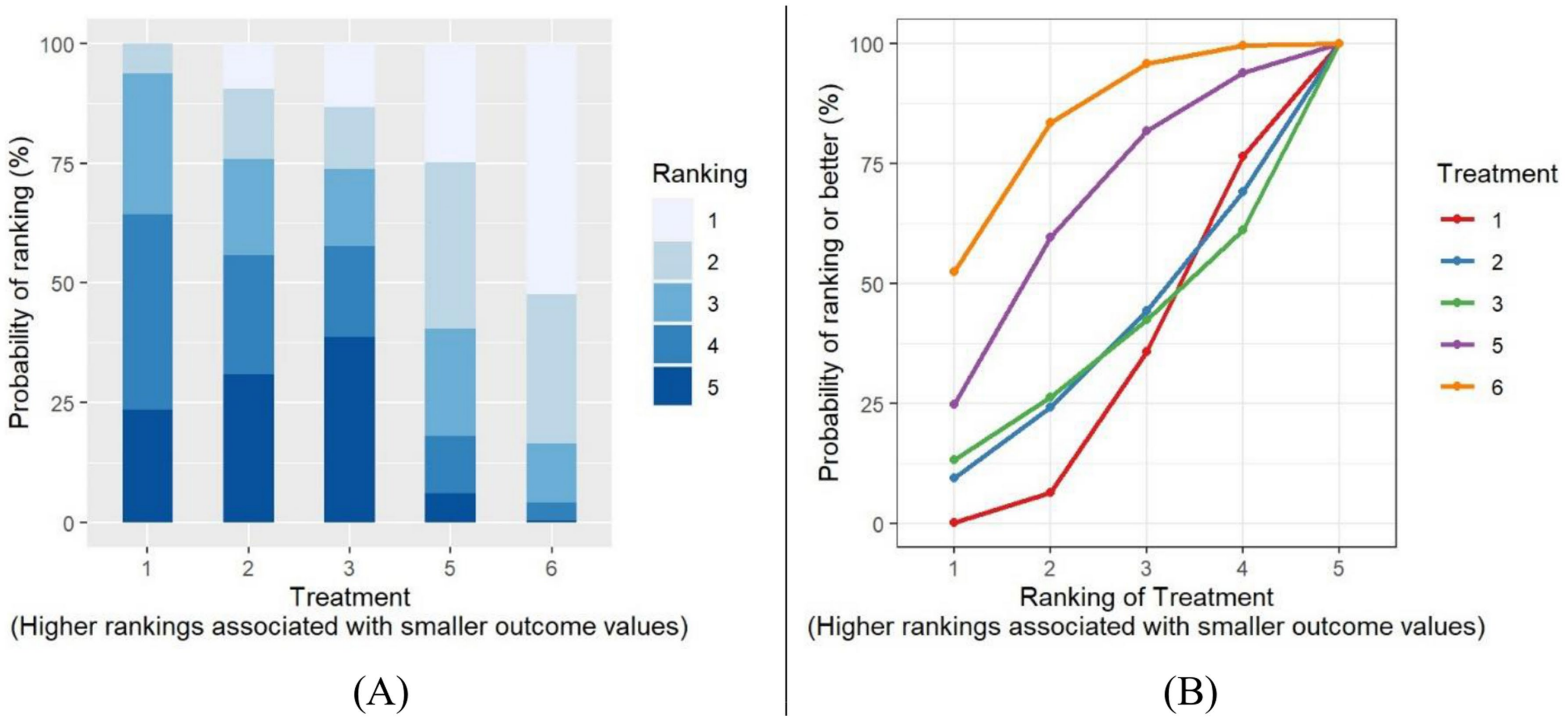
BMIz Plot of treatment rank probabilities **(A)**, BMIz SUCRA plot **(B)**. Treatments (1) control group; (2) PA only; (3) diet & N only; (5) PA & Diet & N; (6) multiple components component.

A treatment rank probability analysis and SUCRA were conducted to determine the ranking probability of each intervention within the network compared to the control group. For BMI, as shown in Figures 6A,B, PA-only interventions emerged as the most effective treatment for reducing BMI, followed by multiple-component interventions. Interestingly, the control group ranked higher than both the PA and Diet and N, and PA and another component interventions. Among all treatments, the PA and another component interventions were identified as the least effective in decreasing BMI. For BMIz, as depicted in Figures 7A,B, multiple-component interventions had the highest probability of being the most effective treatment for reducing BMIz, followed by PA and Diet and N. In contrast, the Diet and N-only group was found to be the least effective in decreasing BMIz.

#### 3.4.4 League plots

**Figure 8:**
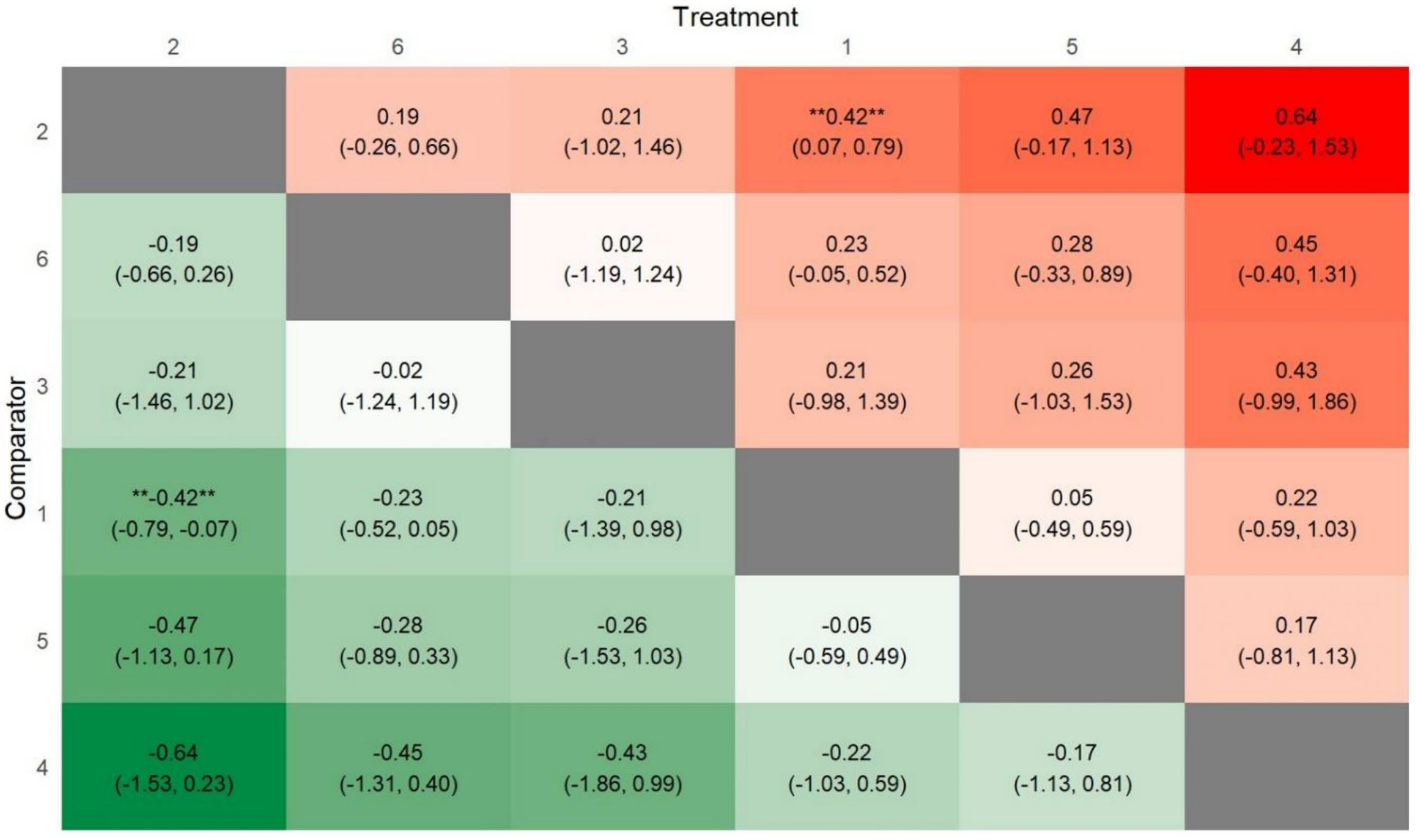
League heat plot for all treatment in the network for BMI comparators (1) control group; (2) PA only; (3) diet & N only; (4) PA & another component; (5) PA & diet & N; (6) multiple components.

**Figure 9:**
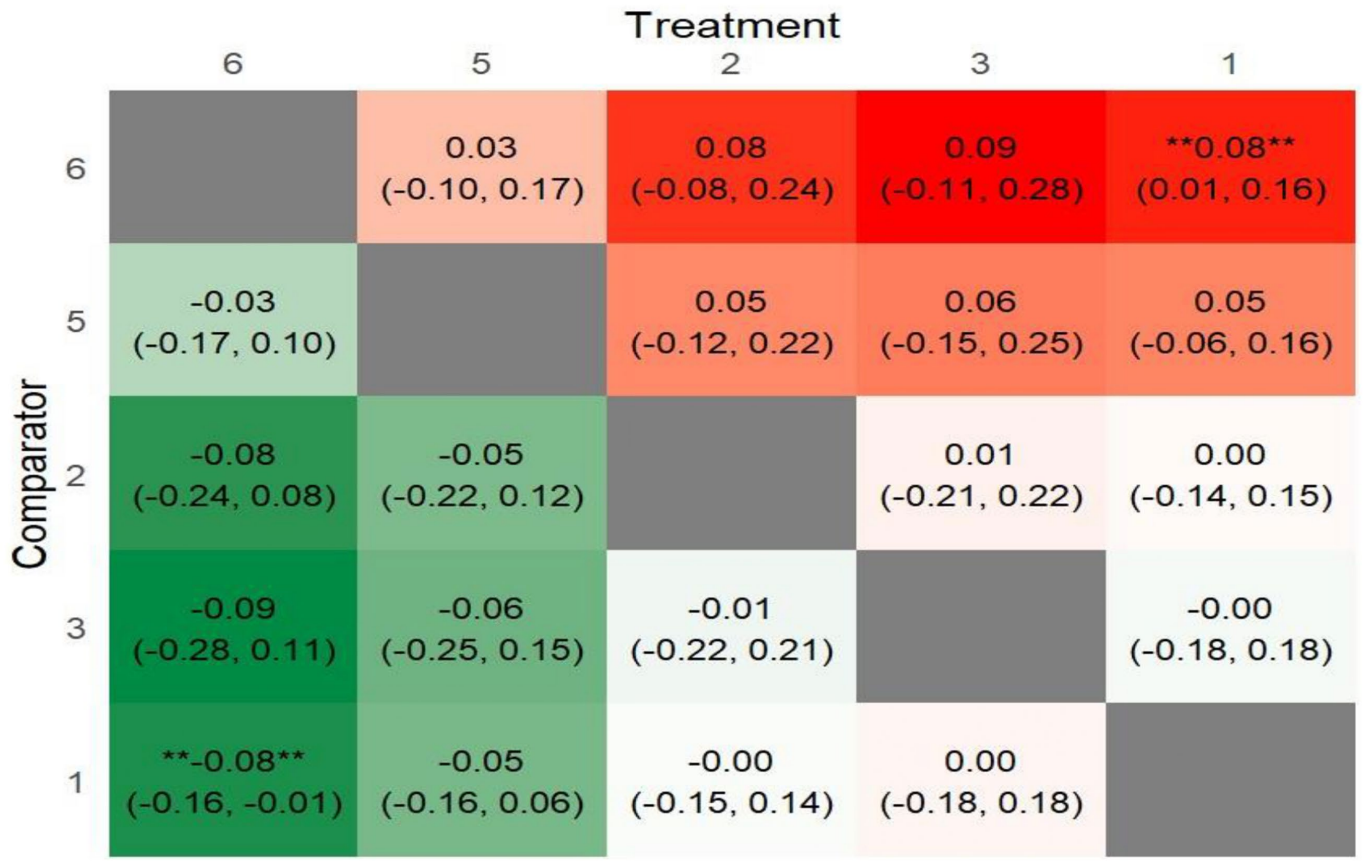
League heat plot for all treatment in the network for BMIz. Comparators (1) control group; (2) PA only; (3) diet & N only; (5) PA & Diet & N; (6) multiple components.

To summarize the NMA results comprehensively, a league plot was created to illustrate the significance of all interventions compared to the control group and other treatments. For BMI, as shown in Figure 8, green cells indicate better performance when comparing treatments. While several interventions appeared effective in reducing BMI, only one—PA-only interventions—demonstrated a statistically significant difference compared to the control group (MD: -0.42, 95% CrI: −0.79, −0.07; *p* = 0.02). For BMIz, as depicted in Figure 9, fewer green cells were observed, indicating a weaker overall effect on BMIz compared to the BMI league plot. Similar to the BMI results, only one intervention—multiple-component interventions—showed a statistically significant difference compared to the control group treatments (MD: −0.08, 95% Crl: −0.16, −0.01; *p* = 0.03).

#### 3.4.5 Forest plots

**Figure 10:**
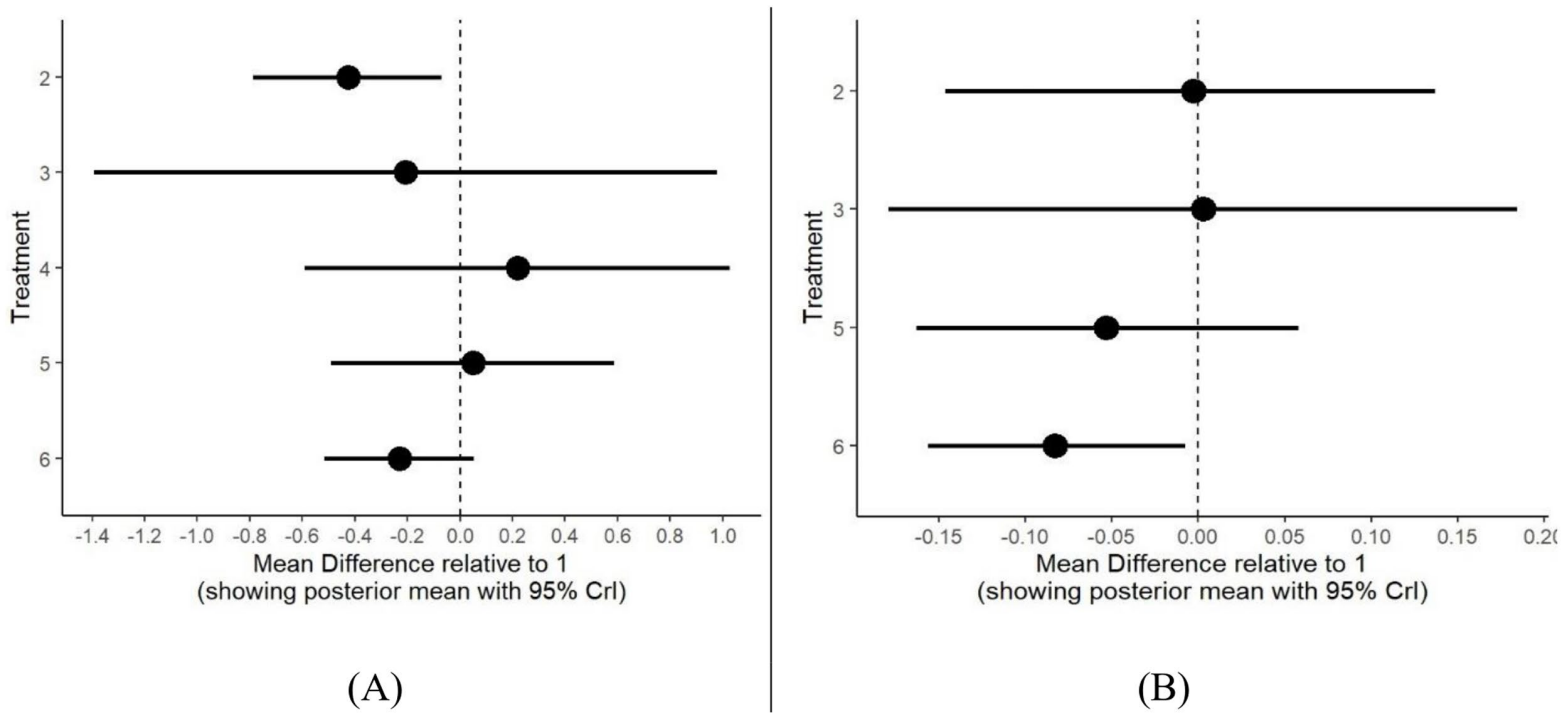
Forest plot for all treatments compared to control group as reference for BMI **(A)** and BMIz **(B)**. Treatments (1) control group; (2) PA only; (3) Diet & N only; (4) PA & another component; (5) PA & diet &N; (6) multiple components.

To provide a clearer visualization of pairwise comparisons among different treatments, forest plots were generated for both BMI and BMIz outcomes. Figure 10 presents a detailed illustration of MD with 95% CrI between interventions. As shown in Figure 10A, PA-only interventions demonstrated the greatest BMI reduction based on MD and 95% CrI when compared to the control group and other interventions. In Figure 10B, multiple-component interventions emerged as the most effective in reducing BMIz compared to the control group and other treatments. It is important to note that the PA and another component interventions were excluded from the BMIz analysis due to a limited amount of supporting evidence in the included studies.

#### 3.4.6 Publication bias

**Figure 11:**
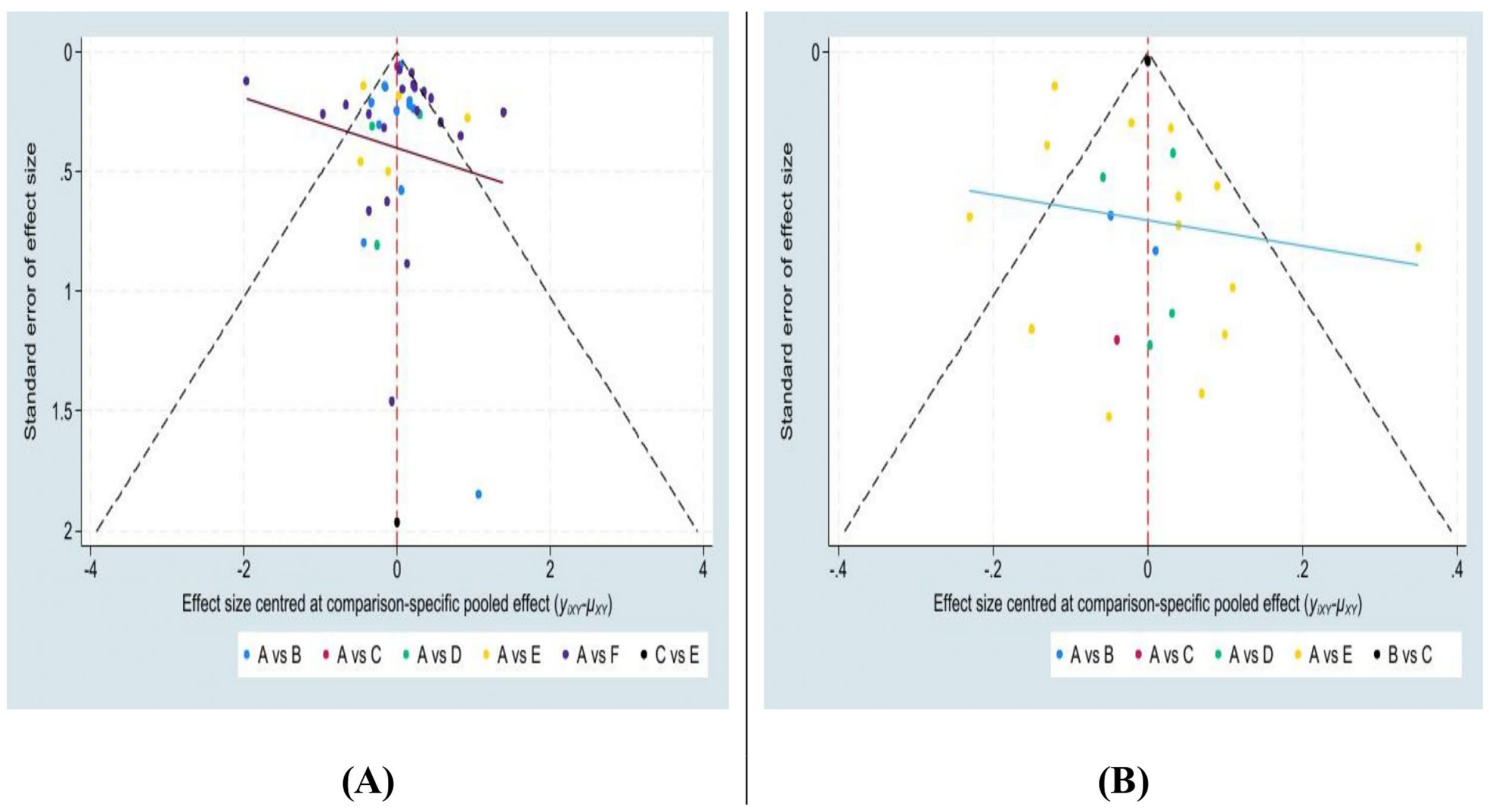
Funnel plots for the effect of intervention arms on BMI **(A)** and BMIz **(B)**.

Funnel plots were used to assess potential publication bias among the included studies. The plots represent the BMI (Figure 11A) and BMIz (Figure 11B). Both plots exhibited a symmetric distribution within the 95% Crl, indicating minimal bias. Although a few outliers were observed, the BMI funnel plot demonstrated a more precise distribution compared to the BMIz plot. Overall, the analysis suggests minimal publication bias or small sample size effects in the included studies.

#### 3.4.7 Sensitivity

**Figure 12:**
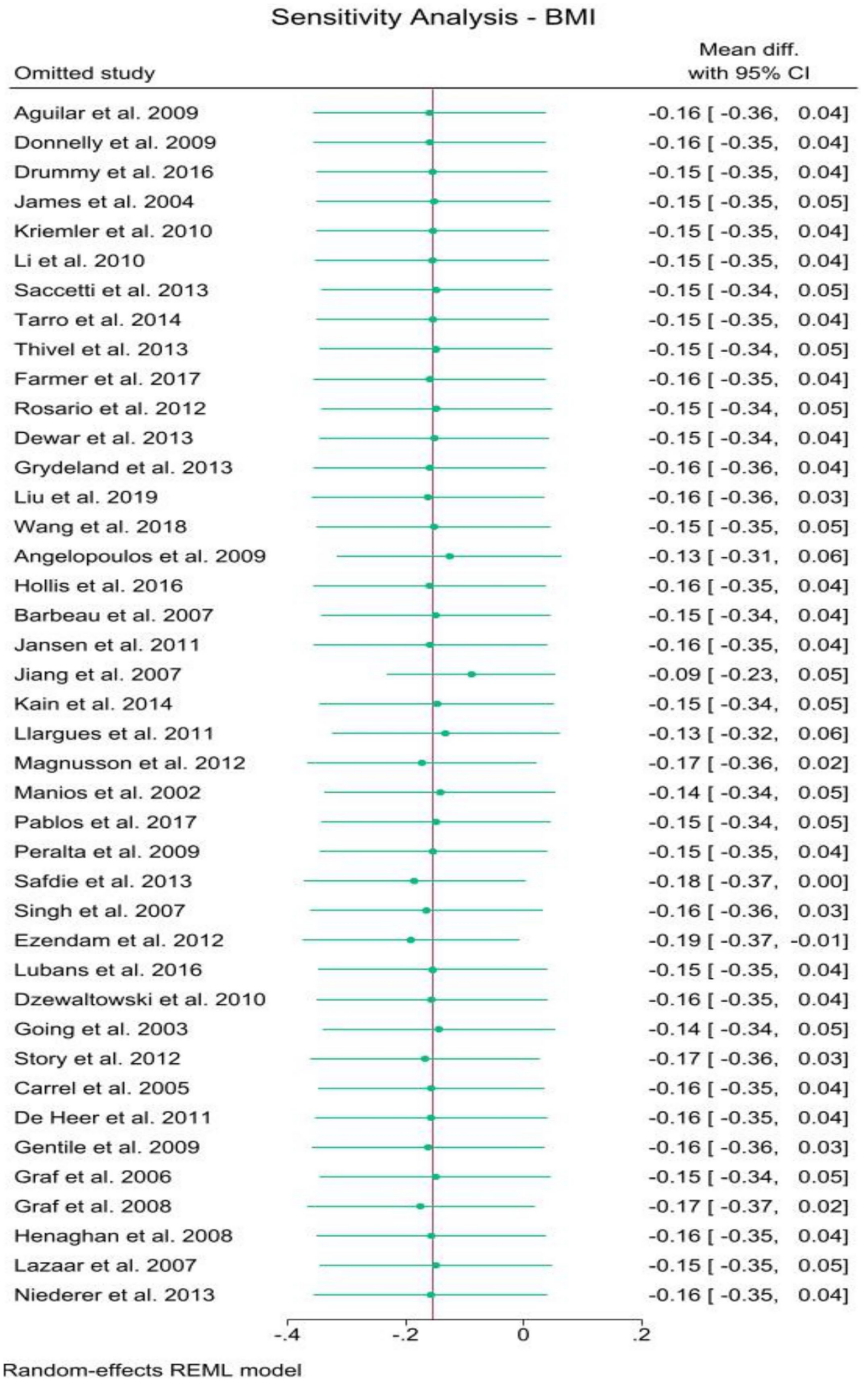
Sensitivity analysis for BMI.

**Figure 13:**
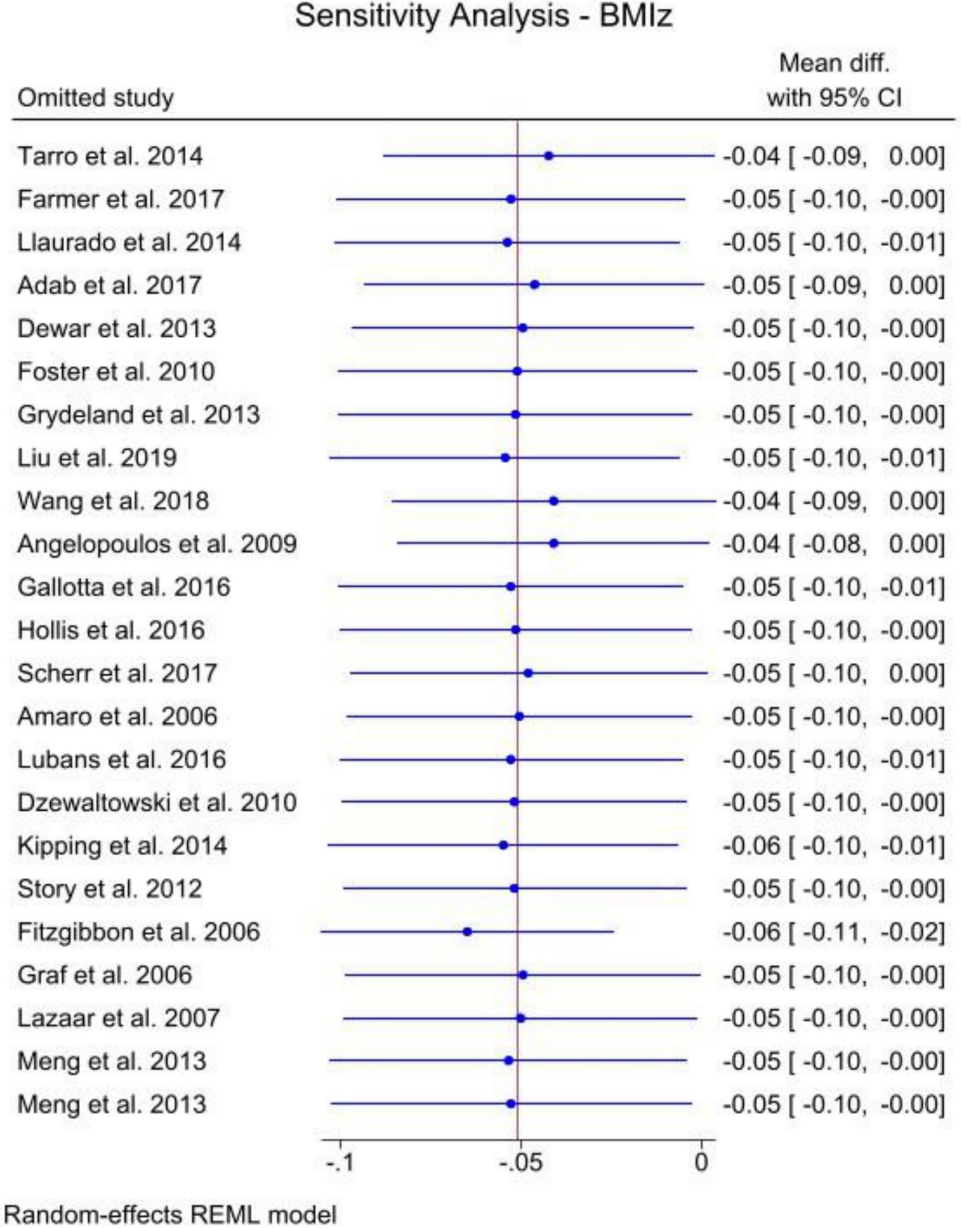
Sensitivity analysis for BMIz.

To evaluate the stability and precision of the results, a leave-one-out sensitivity analysis was performed for both BMI (Figure 12) and BMIz (Figure 13). While a few studies were found to have some influence on the overall estimates, no substantial effects were observed that would significantly impact the results. Both figures demonstrated outcomes within marginal significance, confirming the robustness of the analysis.

## 4 Discussion

This NMA synthesizes current evidence on key factors influencing body composition outcomes in children and adolescents. The primary objective was to consolidate existing data to evaluate the relative effectiveness of various interventions aimed at improving body composition. Specifically, the analysis sought to determine the effectiveness and rank the superiority of different school-based strategies, including PA only, Diet and N only, PA and another component, PA and Diet and N, and multiple-component interventions, in reducing BMI and/or BMIz.

Regarding school-based interventions targeting PA, findings of this NMA suggest that interventions focused exclusively on PA are the most effective in reducing BMI among children and adolescents in school settings. This aligns with previous research, including a meta-analysis of 11 studies that examined differences in physical fitness and body composition between active and sedentary adolescents (108). The results indicated that participation in PA is associated with improved body composition outcomes. However, a previous systematic review and meta-analysis highlighted that the effectiveness of PA interventions may vary depending on their duration and intensity, potentially affecting the consistency of findings (109). Nevertheless, it has been confirmed that PA-only interventions, even with variations in length and intensity, still have a positive impact on reducing BMI and/or BMIz. This assumption aligns with previous research, such as a systematic review of 29 studies evaluating the effectiveness of high-intensity interval training (HIIT) and moderate-intensity continuous training on body composition and cardiorespiratory fitness (110). The results demonstrated positive improvements in BMI values with both types of training. Similar findings were reported in another study (111), which analyzed data from 38 studies involving 1,317 individuals with obesity. This study aimed to rank different PA approaches—including aerobic exercise, resistance training, and HIIT—based on their effectiveness. The analysis concluded that all forms of PA contributed to BMI reduction.

Different PA modalities and intervals seem to have varying levels of effectiveness in improving anthropometric and body composition outcomes. Further research is needed to examine the influence of frequency and the long-term effects of school-based interventions. A meta-analysis evaluated the long-term effects of school-based obesity prevention interventions in children. This review included 19 studies that assessed outcomes more than 12 months post-intervention. The authors concluded that there is no clear evidence of sustained long-term effects on obesity-related outcomes. This suggests that additional factors may play a role in achieving long-term success in preventing obesity (112).

Unsurprisingly, integrating additional components into PA interventions can lead to significant improvements in body composition outcomes. For instance, one study found that combining an exercise intervention with dietary guidance was more effective in improving body shape and BMI compared to exercise alone, diet alone, or no intervention (113). This partially supports the findings of this NMA, as PA interventions combined with diet and nutrition ranked as the second most effective approach in reducing BMIz. Other studies suggest that incorporating even more components, such as parental involvement and energy balance-related behaviors, alongside diet and PA, may further enhance the effectiveness of interventions in improving children’s BMI (114). Consistent with this, several studies have focused on comprehensive, multiple-component interventions to promote healthy weight. A systematic review of 12 studies targeting multiple-component interventions to reduce obesity concluded that such approaches are more effective in reducing BMI compared to PA-only interventions (115). Similarly, another study systematically evaluated the effectiveness of combining a low-calorie diet with cognitive behavioral therapy, meal replacements, or exercise in reducing obesity (116). This analysis included 32 trials with 3,363 participants and found that multiple-component interventions were significantly associated with medium-level weight loss effects. Additionally, a review of 68 RCTs evaluated various interventions for promoting healthy weight in children and adults, concluding that multiple-component approaches and reduced television viewing were the most promising strategies for combating obesity (117). These findings align with the results of this NMA, further validating that multiple-component interventions represent a promising avenue for improving body composition outcomes, as demonstrated by their high ranking in the BMIz analysis.

In summary, while some discrepancies exist in the rankings of interventions based on BMI and BMIz outcomes, interventions focusing solely on PA and those incorporating multiple components appear to be the most effective in improving body composition. Specifically, PA-only interventions ranked first in effectiveness for BMI outcomes, while multiple-component interventions were highly ranked, securing first place for BMI and third for BMIz. However, definitive conclusions about the effectiveness of the multiple-component approach remain elusive, primarily due to the need for greater clarity regarding the specific elements that constitute such interventions.

## 5 Strengths of the study

To date, this is the first NMA designed to evaluate the efficacy and relative rankings of various school-based obesity prevention interventions for improving body composition outcomes. Moreover, this NMA is highly generalizable, as it includes studies from 18 countries, representing diverse cultural and educational contexts. Finally, with a total sample size of 68,489 participants across all included studies, this analysis benefits from a relatively large dataset.

## 6 Limitations of the study

Several limitations were identified in this analysis. First, a number of the included studies were assessed as having a potentially high risk of bias. Second, the scarcity of studies supporting the “PA and another component” intervention arm led to its exclusion from the BMIz analysis. Third, the intensity and duration of PA interventions were not thoroughly examined, which may have introduced heterogeneity across studies. Additionally, while the “multiple components” approach appeared to be among the most effective for improving body composition outcomes, the results are potentially compromised by varying definitions of “multiple components” across studies. Due to the lack of standardized classifications for these components, these findings should be interpreted with caution. Finally, light PA, which might also contribute to BMI or BMIz, was not explored as part of PA in the current study (118, 119).

## 7 Areas for further research

While PA-only and multiple-component interventions appear to be the most effective approaches, further systematic reviews are needed to examine each intervention type independently. Additional research is crucial to validate PA-only interventions, specifically regarding their intensity and duration. Moreover, future reviews are recommended to explore and standardize the classification of multiple-component interventions.

## 8 Conclusion

In conclusion, school-based obesity prevention interventions focusing solely on PA or utilizing multiple components have demonstrated positive effects on both BMI and BMIz. However, careful attention should be given to the specifics of PA programs, such as their duration, intensity, and modality. Additionally, evaluating multiple-component programs requires caution, as their content can vary significantly across studies. To address this variability, establishing a standardized classification for the components of multiple-component interventions is highly recommended.

## Data Availability

The original contributions presented in the study are included in the article/Supplementary material, further inquiries can be directed to the corresponding author.
