# Supplementary material for: Comparative effectiveness of school-based obesity prevention programs for children and adolescents: a systematic review and network meta-analysis
Source: Front Public Health. 2024 Dec 17;12:1504279. doi: 10.3389/fpubh.2024.1504279 (PMC11685220; doi:10.3389/fpubh.2024.1504279)
Supplement: Supplementary file 1 [file Supplementary_file_1.docx]

**Supplementary file 1. Search Strategy**

| MEDLINE (PubMed) |
| --- |
| 1. Child[MeSH] OR Pediatrics[MeSH] OR "Pediatric Obesity"[MeSH] OR child*[TIAB] OR "pre-school*" [TIAB] OR preschool* [TIAB] OR "elementary school" [TIAB] OR "kid*" [TIAB] OR youth* [TIAB] OR pediatric* [TIAB] 2. Obesity[MeSH] OR "Obesity Management"[MeSH] OR "Obesity, Abdominal"[MeSH] OR Overweight[MeSH] OR "Weight Loss"[MeSH] OR adiposity[TIAB] OR obes*[TIAB] OR overweight[TIAB] 3. (clinical[TIAB] AND trial[TIAB]) OR Clinical Trials as Topic[MeSH Terms] OR clinical trial[Publication Type] OR random*[TIAB] OR Random Allocation[MeSH Terms] OR randomly[TIAB] 4. Exercise[MeSH] OR "Exercise Therapy"[MeSH] OR "Motor Activity"[MeSH] OR "Physical Education and Training"[MeSH] OR exercise[TIAB] OR "physical activity"[TIAB] OR "physical activities"[TIAB] 5. Diet[MeSH] OR "Diet, Food, and Nutrition"[MeSH] OR "Diet Therapy"[MeSH] OR diet*[TIAB] OR food[TIAB] OR nutrition[TIAB] 6. "Behavior Therapy"[MeSH] OR "Counseling"[MeSH] OR "Health Education"[MeSH] OR "Patient Education as Topic"[MeSH] OR "behavior therapy"[TIAB] OR "behavioral therapy"[TIAB] 7. "Life Style"[MeSH] OR "Healthy Lifestyle"[MeSH] OR "Health Behavior"[MeSH] OR "lifestyle"[TIAB] OR "health behavior"[TIAB] OR "health related behavior*"[TIAB] 8. ((Child[MeSH] OR Pediatrics[MeSH] OR "Pediatric Obesity"[MeSH] OR child*[TIAB] OR "pre-school*" [TIAB] OR preschool* [TIAB] OR "elementary school" [TIAB] OR "kid*" [TIAB] OR youth* [TIAB] OR pediatric* [TIAB]) AND (Obesity[MeSH] OR "Obesity Management"[MeSH] OR "Obesity, Abdominal"[MeSH] OR Overweight[MeSH] OR "Weight Loss"[MeSH] OR adiposity[TIAB] OR obes*[TIAB] OR overweight[TIAB])) AND ((clinical[TIAB] AND trial[TIAB]) OR Clinical Trials as Topic[MeSH Terms] OR clinical trial[Publication Type] OR random*[TIAB] OR Random Allocation[MeSH Terms] OR randomly[TIAB]) 9. (((Child[MeSH] OR Pediatrics[MeSH] OR "Pediatric Obesity"[MeSH] OR child*[TIAB] OR "pre-school*" [TIAB] OR preschool* [TIAB] OR "elementary school" [TIAB] OR "kid*" [TIAB] OR youth* [TIAB] OR pediatric* [TIAB]) AND (Obesity[MeSH] OR "Obesity Management"[MeSH] OR "Obesity, Abdominal"[MeSH] OR Overweight[MeSH] OR "Weight Loss"[MeSH] OR adiposity[TIAB] OR obes*[TIAB] OR overweight[TIAB])) AND ((clinical[TIAB] AND trial[TIAB]) OR Clinical Trials as Topic[MeSH Terms] OR clinical trial[Publication Type] OR random*[TIAB] OR Random Allocation[MeSH Terms] OR randomly[TIAB])) AND (Exercise[MeSH] OR "Exercise Therapy"[MeSH] OR "Motor Activity"[MeSH] OR "Physical Education and Training"[MeSH] OR exercise[TIAB] OR "physical activity"[TIAB] OR "physical activities"[TIAB]) 10. (((Child[MeSH] OR Pediatrics[MeSH] OR "Pediatric Obesity"[MeSH] OR child*[TIAB] OR "pre-school*" [TIAB] OR preschool* [TIAB] OR "elementary school" [TIAB] OR "kid*" [TIAB] OR youth* [TIAB] OR pediatric* [TIAB]) AND (Obesity[MeSH] OR "Obesity Management"[MeSH] OR "Obesity, Abdominal"[MeSH] OR Overweight[MeSH] OR "Weight Loss"[MeSH] OR adiposity[TIAB] OR obes*[TIAB] OR overweight[TIAB])) AND ((clinical[TIAB] AND trial[TIAB]) OR Clinical Trials as Topic[MeSH Terms] OR clinical trial[Publication Type] OR random*[TIAB] OR Random Allocation[MeSH Terms] OR randomly[TIAB])) AND (Diet[MeSH] OR "Diet, Food, and Nutrition"[MeSH] OR "Diet Therapy"[MeSH] OR diet*[TIAB] OR food[TIAB] OR nutrition[TIAB]) 11. (((Child[MeSH] OR Pediatrics[MeSH] OR "Pediatric Obesity"[MeSH] OR child*[TIAB] OR "pre-school*" [TIAB] OR preschool* [TIAB] OR "elementary school" [TIAB] OR "kid*" [TIAB] OR youth* [TIAB] OR pediatric* [TIAB]) AND (Obesity[MeSH] OR "Obesity Management"[MeSH] OR "Obesity, Abdominal"[MeSH] OR Overweight[MeSH] OR "Weight Loss"[MeSH] OR adiposity[TIAB] OR obes*[TIAB] OR overweight[TIAB])) AND ((clinical[TIAB] AND trial[TIAB]) OR Clinical Trials as Topic[MeSH Terms] OR clinical trial[Publication Type] OR random*[TIAB] OR Random Allocation[MeSH Terms] OR randomly[TIAB])) AND ("Behavior Therapy"[MeSH] OR "Counseling"[MeSH] OR "Health Education"[MeSH] OR "Patient Education as Topic"[MeSH] OR "behavior therapy"[TIAB] OR "behavioral therapy"[TIAB]) 12. (((Child[MeSH] OR Pediatrics[MeSH] OR "Pediatric Obesity"[MeSH] OR child*[TIAB] OR "pre-school*" [TIAB] OR preschool* [TIAB] OR "elementary school" [TIAB] OR "kid*" [TIAB] OR youth* [TIAB] OR pediatric* [TIAB]) AND (Obesity[MeSH] OR "Obesity Management"[MeSH] OR "Obesity, Abdominal"[MeSH] OR Overweight[MeSH] OR "Weight Loss"[MeSH] OR adiposity[TIAB] OR obes*[TIAB] OR overweight[TIAB])) AND ((clinical[TIAB] AND trial[TIAB]) OR Clinical Trials as Topic[MeSH Terms] OR clinical trial[Publication Type] OR random*[TIAB] OR Random Allocation[MeSH Terms] OR randomly[TIAB])) AND ("Life Style"[MeSH] OR "Healthy Lifestyle"[MeSH] OR "Health Behavior"[MeSH] OR "lifestyle"[TIAB] OR "health behavior"[TIAB] OR "health related behavior*"[TIAB]) |
| Embase |
| #1  'child'/exp OR 'child' OR 'children' OR 'pediatric'/exp OR 'preschool'/exp OR 'primary school'/exp OR 'elementary school' OR 'primary school' OR 'juvenile'/exp OR 'juvenile' OR 'youth'  #2  'obesity'/exp OR 'adipose tissue hyperplasia' OR 'adipositas' OR 'adiposity' OR 'alimentary obesity' OR 'body weight, excess' OR 'corpulency' OR 'fat overload syndrome' OR 'nutritional obesity' OR 'obesitas' OR 'obesity' OR 'overweight' OR 'childhood obesity'/exp OR 'childhood obesity' OR 'paediatric obesity' OR 'pediatric obesity'  #3  'exercise'/exp OR 'biometric exercise' OR 'effort' OR 'exercise' OR 'exercise capacity' OR 'exercise performance' OR 'exercise training' OR 'exertion' OR 'fitness training' OR 'fitness workout' OR 'physical conditioning, human' OR 'physical effort' OR 'physical exercise' OR 'physical exertion' OR 'physical work-out' OR 'physical workout' OR 'physical activity'/exp OR 'activity, physical' OR 'physical activity' OR 'physical education'/exp OR 'adapted physical education' OR 'education, physical' OR 'physical education' OR 'physical education and training'  #4  'diet'/exp OR 'diet' OR 'diet influence' OR 'diet regimen' OR 'diet surveys' OR 'dietary effect' OR 'dietary influence' OR 'dietary survey' OR 'dietary surveys' OR 'dieting' OR 'food'/exp OR 'food' OR 'food and beverages' OR 'nutrition'/exp OR 'diet, food, and nutrition' OR 'nutrition' OR 'nutrition council' OR 'nutrition physiology' OR 'nutrition processes' OR 'nutrition research' OR 'nutrition study' OR 'nutrition survey' OR 'nutritional physiology' OR 'nutritive solution'  #5  'behavior therapy'/exp OR 'behavior psychotherapy' OR 'behavior therapy' OR 'behavior training' OR 'behavior treatment' OR 'behavioral psychotherapy' OR 'behavioral therapy' OR 'behaviour psychotherapy' OR 'behaviour therapy' OR 'behaviour training' OR 'behaviour treatment' OR 'behavioural psychotherapy' OR 'behavioural therapy' OR 'therapy, behavior' OR 'therapy, behaviour' OR 'treatment, behavior' OR 'treatment, behaviour' OR 'counseling'/exp OR 'counseling' OR 'counselling' OR 'health education'/exp OR 'education, health' OR 'health education' OR 'health fairs' OR 'health science education' OR 'health sciences education'  #6  'lifestyle modification'/exp OR 'life style change' OR 'life style changes' OR 'life style modification' OR 'life style modifications' OR 'lifestyle change' OR 'lifestyle changes' OR 'lifestyle modification' OR 'lifestyle modifications' OR 'health behavior'/exp OR 'behavior, health' OR 'behaviour, health' OR 'health behavior' OR 'health behaviour' OR 'health promoting behavior' OR 'health promoting behaviour' OR 'health related behavior' OR 'health related behaviour' OR 'lifestyle'/exp OR 'life style' OR 'lifestyle'  #7  'body composition'/exp OR 'body composition' OR 'composition, body' OR 'body mass'/exp OR 'bmi (body mass index)' OR 'body mass' OR 'body mass index' OR 'body fat percentage'/exp OR '%bf (body fat)' OR 'body fat percent' OR 'body fat percentage' OR 'bodyfat percentage' OR 'percent body fat' OR 'percentage of body fat' OR 'percentage of bodyfat'  #8  'randomized controlled trial'/exp OR 'controlled trial, randomized' OR 'randomised controlled study' OR 'randomised controlled trial' OR 'randomized controlled study' OR 'randomized controlled trial' OR 'trial, randomized controlled'  #9  #1 AND #2 AND #7 AND #8  #10  #3 AND #9 AND [embase]/lim AND [article]/lim AND [english]/lim AND ([preschool]/lim OR [school]/lim)  #11  #4 AND #9 AND [embase]/lim AND [article]/lim AND [english]/lim AND ([preschool]/lim OR [school]/lim)  #12  #5 AND #9 AND [embase]/lim AND [article]/lim AND [english]/lim AND ([preschool]/lim OR [school]/lim)  #13  #6 AND #9 AND AND [embase]/lim AND [article]/lim AND [english]/lim AND ([preschool]/lim OR [school]/lim) |
| CENTRAL |
| physical activity OR exercise OR training OR activities OR fitness in Title Abstract Keyword AND nutrition OR diet OR obesity OR weight loss in Title Abstract Keyword AND adiposity OR body mass index OR anthropometric OR BMI OR obesity in Title Abstract Keyword AND school children OR school program OR school based intervention OR randomized controlled trials in Title Abstract Keyword NOT animals in All Text - in Trials |
| CINAHL  Expanders - Apply related words; Also search within the full text of the articles; Apply equivalent subjects  Search modes - Boolean/Phrase |
| S1  AB (children or kids or youth or child or childhood or school age or preschool or pediatric or elementary school or primary school or kid or juvenile or parent)  S2  AB (obesity or overweight or fat or obese or unhealthy weight or adiposity or childhood obesity or pediatric obesity or high bmi or excess body weight)  S3  AB (randomized controlled trials or rct or randomised control trials or randomized evaluation or randomized trials or randomized or clinical trial or clinical trials)  S4  AB (exercise or physical fitness or physical activity or exercise therapy or physical education or exercise training or fitness training or fitness workout or physical workout or physical education and training)  S5  AB (diet or nutrition or food habit or eating habit or food or dietary intake or diet therapy or diet regimen or dietary effect or food and beverages)  S6  AB (behavioral therapy or behavioral interventions or behavior modification or counseling or health behavior or psychotherapy or behavior treatment or behaviour therapy or behaviour treatment or behavioural therapy or health education or health promotion)  S7  AB (lifestyle changes or lifestyle modification or lifestyle choices or lifestyle change or intervention or life style or health related behaviour)  S8  S1 AND S2 AND S3  S9  S4 AND S8  S10  S5 AND S8  S11  S6 AND S8  S12  S7 AND S8 |
