# Supplementary material for: Comparative effectiveness of school-based obesity prevention programs for children and adolescents: a systematic review and network meta-analysis
Source: Front Public Health. 2024 Dec 17;12:1504279. doi: 10.3389/fpubh.2024.1504279 (PMC11685220; doi:10.3389/fpubh.2024.1504279)
Supplement: Supplementary file 3 [file Supplementary_file_3.docx]

|  | | | Table 1. Cochrane Risk of Bias Assessment for Randomised Controlled Trials (low, high, unclear) | | | | | | | | | | | | | | |
| --- | --- | --- | --- | --- | --- | --- | --- | --- | --- | --- | --- | --- | --- | --- | --- | --- | --- |
| **No.** | | **Study** | | | **Random sequence generation** | | **Allocation concealment** | | **Blinding of Participants and personnel** | | **Blinding of outcome assessment** | | **Incomplete outcome data addressed** | | **Selective reporting** | |  |
|  | | Aguilar et al. | | | Low | | Low | | High | | Low | | Low | | Low | |  |
|  | | Donnelly et al. | | | Low | | Unclear | | Low | | Low | | Low | | Low | |  |
|  | | Drummy et al. | | | Unclear | | Unclear | | Unclear | | Unclear | | Low | | Low | |  |
|  | | James et al. | | | Low | | Low | | Low | | Unclear | | Low | | Low | |  |
|  | | Kriemler et al. | | | Low | | Low | | Low | | Low | | Low | | Low | |  |
|  | | Li et al. | | | Low | | Low | | Low | | Low | | Low | | Low | |  |
|  | | Saccetti et al. | | | Low | | Low | | Unclear | | Unclear | | Low | | Low | |  |
|  | | Tarro et al. | | | High | | High | | High | | Low | | Low | | Low | |  |
|  | | Thivel et al. | | | Low | | Low | | Low | | Low | | Low | | Low | |  |
|  | | Farmer et al. | | | Low | | Low | | Low | | Low | | Low | | Low | |  |
|  | | Llaurado et al. | | | Low | | Unclear | | Unclear | | High | | Low | | Low | |  |
|  | | Rosario et al. | | | Low | | Unclear | | Unclear | | High | | Low | | Low | |  |
|  | | Adab et al. | | | Low | | Low | | Low | | Low | | Low | | Low | |  |
|  | | Dewar et al. | | | Low | | Unclear | | Unclear | | Low | | Low | | Low | |  |
|  | | Foster et al. | | | Low | | Low | | Low | | Low | | Low | | Low | |  |
|  | | Grydeland | | | Low | | High | | High | | Low | | Low | | Low | |  |
|  | | Liu et al. | | | Low | | Low | | Low | | Low | | Low | | Low | |  |
|  | | Wang et al. | | | Low | | Low | | Low | | Low | | Low | | Low | |  |
|  | | Angelopoulos et al. | | | Low | | Unclear | | Unclear | | Unclear | | Unclear | | Low | |  |
|  | | Gallotta et al. | | | Low | | Low | | High | | High | | High | | Low | |  |
|  | | Hollis et al. | | | Low | | Low | | Low | | Low | | Low | | Low | |  |
|  | | Scherr et al. | | | Low | | Unclear | | Unclear | | Unclear | | Low | | Low | |  |
|  | | Barbeau et al. | | | High | | High | | High | | High | | Low | | Low | |  |
|  | | Jansen et al. | | | Low | | Unclear | | Unclear | | Unclear | | Low | | Low | |  |
|  | | Jiang et al. | | | Low | | Unclear | | Unclear | | Unclear | | Low | | Low | |  |
|  | | Kain et al. | | | Low | | Unclear | | Unclear | | Unclear | | Low | | Low | |  |
|  | | Llargues et al. | | | Low | | Low | | Unclear | | Unclear | | Low | | Low | |  |
|  | | Magnusson et al. | | | Low | | Unclear | | Unclear | | Unclear | | Low | | Low | |  |
|  | | Manios et al. | | | Low | | Low | | Unclear | | Unclear | | Low | | Low | |  |
|  | | Pablos et al. | | | Low | | Low | | Low | | Unclear | | Low | | Low | |  |
|  | | Peralta et al. | | | Low | | Unclear | | Unclear | | Unclear | | Low | | Low | |  |
|  | | Safdie et al. | | | High | | High | | High | | High | | Low | | Low | |  |
|  | | Singh et al. | | | Low | | High | | High | | High | | High | | Low | |  |
|  | | Amaro et al. | | | Low | | Unclear | | Unclear | | Unclear | | Low | | Low | |  |
|  | | Ezendam et al. | | | Low | | Unclear | | Unclear | | Unclear | | Low | | Low | |  |
|  | | Lubans et al. | | | Low | | Low | | Low | | Low | | Unclear | | Low | |  |
|  | | Pfeiffer et al. | | | Low | | Unclear | | Unclear | | Unclear | | Low | | Low | |  |
|  | | Crouter et al. | | | Low | | Low | | Unclear | | Unclear | | Low | | Low | |  |
|  | | Dzewaltowski et al. | | | Low | | Low | | Low | | Low | | Low | | Low | |  |
|  | | Going et al. | | | Unclear | | Unclear | | High | | Unclear | | Low | | Low | |  |
|  | | Kipping et al. | | | Low | | Low | | Low | | Low | | Low | | Low | |  |
|  | | Story et al. | | | Low | | Unclear | | Unclear | | Unclear | | Low | | Low | |  |
|  | | Carrel et al. | | | Unclear | | Unclear | | Unclear | | Unclear | | Low | | Low | |  |
|  | | Crespo et al. | | | Low | | Unclear | | Unclear | | Low | | Low | | Low | |  |
|  | | de Heer et al. | | | Low | | High | | High | | High | | Low | | Low | |  |
|  | | Fitzgibbon et al. | | | Low | | High | | High | | High | | Low | | Low | |  |
|  | | Gentile et al. | | | Low | | Unclear | | High | | High | | Low | | Low | |  |
|  | | Graf et al. | | | Low | | Unclear | | High | | High | | Low | | Low | |  |
|  | | Graf et al. | | | Low | | Unclear | | High | | High | | Low | | Low | |  |
|  | | Henaghan et al. | | | Low | | Unclear | | Low | | Unclear | | Low | | Low | |  |
|  | | Lazaar et al. | | | Unclear | | Unclear | | Unclear | | Unclear | | Low | | Low | |  |
|  | | Meng et al. | | | Low | | Unclear | | Unclear | | Unclear | | Low | | Low | |  |
|  | | Niederer et al. | | | Low | | Low | | Low | | Low | | Low | | Low | |  |

Low risk = described adequately within the study; Unclear risk = described somewhat adequately within the study; High risk = was poorly described or not described within the study.
